# The Evolutionary and Developmental Foundations of Mathematics

**DOI:** 10.1371/journal.pbio.0060019

**Published:** 2008-02-05

**Authors:** Michael J Beran

## Abstract

Recent behavioral and neuroimaging studies with humans and monkeys provide compelling evidence of shared numerical capacities across species. Our understanding of the emergence of human mathematical competence is well-served by these kinds of comparative assessments.

Understanding the evolutionary precursors of human mathematical ability is a highly active area of research in psychology and biology with a rich and interesting history. At one time, numerical abilities, like language, tool use, and culture, were thought to be uniquely human. However, at the turn of the 20th century, scientists showed more interest in the numerical abilities of animals. The earliest research was focused on whether animals could count in any way that approximated the counting skills of humans [1,2], though many early studies lacked the necessary scientific controls to truly prove numerical abilities in animals. In addition, both the public and many in the scientific community too readily accepted cases of “genius” animals, including those that performed amazing mathematical feats. One such animal still lends its name to the phenomenon of inadvertent cuing of animals by humans: Clever Hans. Hans was a horse that seemed to calculate solutions to all types of numerical problems. In reality, the horse was highly attuned to the subtle and inadvertent bodily movements that people would make when Hans had reached the correct answer (by tapping his hoof) and should have stopped responding [3]. One consequence of this embarrassing realization was a backlash for the better part of the 20th century against the idea that animals could grasp numerical concepts. The second, more positive consequence, however, was that future researchers would include appropriate controls to account for such cues.

A resurgence of interest in animal numerical abilities in the early 1980s followed closely on the heels of a landmark book on children's number learning that outlined the critical principles that children must master to become proficient in counting [4]. This resurgence also was the result of a general increase in studies of animal cognition and intelligence. New research programs provided compelling evidence that animals are sensitive to the numerical properties of various kinds of things, even if they do not quite reach the level of human counting abilities. For instance, multiple research teams showed that chimpanzees could learn the meanings of Arabic numerals when taught to collect sets of items to match the values of numerals [5,6], to label sets of items with the correct numeral [7,8], and even to add the values of those numerals [9]. Animal numerical competence had reclaimed the spotlight, and it remains a highly visible area of animal cognition research. Today, we know just how important quantity and number concepts are for a great variety of animals such as salamanders [10], rats [11], various types of birds [12–14], dolphins [15], monkeys [16,17], and apes [18,19].

What consistently emerges from these kinds of studies is that animals are mathematically inclined, but only to a certain degree. Their performance usually is constrained by an objective measure of task difficulty that relates to well-known psychophysical phenomena. Namely, animals seem to use approximate representations of number. For example, in comparison tasks, performance can nearly always be predicted very well by knowing the relation of the two sets to each other. As the difference (or quantitative distance) between sets becomes smaller, the task of choosing the correct set becomes harder (for example, comparing 4 to 6 is easier than comparing 4 to 5). When the difference between sets is held constant, then the task becomes harder as both sets increase in their overall magnitude (for example, comparing 4 to 6 is easier than comparing 6 to 8). What is interesting is that when adult humans are prevented from counting, they also show similar evidence that they have access to a system of noisy magnitudes that they use as a form of nonverbal representation [20,21], and even societies without language-based numerical systems show evidence of nonverbal number approximation [22,23]. In fact, when directly compared, monkeys often show highly similar patterns of performance to young human children [24] and human adults [25–27]. The latest example of such similarities in performance comes from a paper in *PLoS Biology* by Cantlon and Brannon [28]. Researchers tested humans and monkeys in a task where two sets of dots were shown in succession on a computer screen, and participants had to add the sets and then find a match option that had the same total number of dots. Humans did better than monkeys, but the important finding was that both species were constrained in their performance based on how closely the two options resembled each other. The researchers concluded that monkeys and humans share components of a mathematical tool kit that can be applied to various types of problems. The novel aspect of this research is that it shows that monkeys, like humans, can add sets together and remember the total number of items. Most likely, monkeys are not the only animals to share these abilities with humans.

Given these behavioral similarities, one wonders whether monkeys and humans might not only perform these tasks at similar levels, but also in truly similar ways. Hypothetical models for numerical representation can account for the distance and magnitude effects described above [29–31], and environmental pressures seem to place a premium on an approximate “number sense” [32] for nonhuman animals as well as humans. Research with neuropsychological patients and from functional neuroimaging studies indicates that two brain areas, the intraparietal sulcus (IPS) and the prefrontal cortex (PFC), seem intricately linked to number skills in humans (for overviews, see [32,33]). We now know that animal brains also are attuned to numerical properties. There is evidence that there are distinct neural populations and processing stages within the IPS for different quantity presentation types (e.g., sequential versus simultaneous) in monkeys, although abstract representations that occur later in processing may subsume these distinct stages [34]. Single neurons in the ventral intraparietal region (VIP) and PFC in the macaque brain have been found to be attuned to specific numerical values [35,36]. Thus, a neuron in VIP responds maximally to one value, and the firing rate decreases with distance from this preferred value. However, a recent paper by Roitman, Brannon, and Platt published in *PLoS Biology* shows that another region in the parietal cortex, the lateral intraparietal region, encodes numbers differently than the VIP [37]. These neurons increase or decrease in activity based on the number of elements in a visual array, suggesting that they serve to represent accumulated magnitude, an important part of the formal counting routine that provides cardinal (exact) values of numerosity. They may even provide the magnitude information necessary for other brain regions to discern cardinal numerical representations as the brain moves from summing and estimating magnitude to representing exact numerical information. Thus, these data are exciting because they provide a link between theoretical models of number processing and actual brain/behavior relations.

Human mathematical abilities, of course, are highly dependent on symbolic representations of number. A recent paper by Diester and Nieder published in *PLoS Biology* shows that brain areas critical to processing symbolic and analogue numerosities in humans also support numerical processing in monkeys [38]. After monkeys learned to associate Arabic numerals with specific numbers of items, the researchers recorded from single neurons in the PFC and IPS when monkeys judged whether two successive analog arrays were the same in number or whether an analog array matched a numeral in a pairing. PFC neurons were selectively responsive to given numerical values, presented in either analog or symbolic formats. In other words, the PFC in monkeys seems to be involved in the association between symbols and numerical concepts, and it builds upon the capacities of the IPS to encode approximate numerical information early in quantity processing. By four years of age, the IPS in human children is already responsive to changes in the numerosity of visual arrays [39], but the parietal cortex shows a more protracted developmental trajectory for the representation of symbolic numbers. Specifically, children who have not yet become proficient with numerals show elevated PFC activity in response to numerals, whereas parietal areas seemingly take over as proficiency with symbols emerges [40,41]. In adult humans, representation of numerical information across many formats (numerals, analog stimuli, number words) relies substantially on parietal areas [42].

A new report in *PLoS Biology* by Izard, Dehaene-Lambertz, and Dehaene [43] using event-related potentials also supports the idea that humans are born with a dedicated system for numerical processing. Three-month-old children watched as a stream of images consisting of discrete numbers of items was presented. Usually, the number and type of items stayed consistent across images, but the critical data came from trials in which the number of items changed or the type of items changed while the number stayed the same. Different parts of the brain responded to these types of changes, with the prefronto-parietal pathway again responsive to changes in numerosity. This, too, supports the idea that human brains are attuned early in development to number as a highly relevant dimension of the external world.

The Izard et al. paper also is important for what it did not show: differential cerebral responses to small versus large numbers of items. A recent debate within the numerical cognition literature pertains to whether different mechanisms support the representation of small sets and large sets by animals and young children. This *two core number systems hypothesis* [44] draws some support from studies both with monkeys [45] and young children [46,47], although other studies show no such distinction in the processing of small sets and large sets [25,27,48,49]. The data from Izard et al. support the idea that human infants and nonhuman primates share the ability for analog representation across a wide range of numerosities through use of a singular mechanism rather than two distinct mechanisms.

These recent studies have expanded our understanding of the evolution of numerical cognition. Brains (both human and nonhuman) have evolved to deal with numerosity, with different regions supporting different mechanisms of numerical representation. These phylogenetically widespread capacities seem perfectly suited to support survival. Any creature that can tell the difference between a tree with 10 pieces of fruit from another with only six pieces, or between two predators and three on the horizon, has a better chance of surviving and reproducing. At the same time, telling the difference between 24 and 28 pieces of fruit (or nine predators versus 10) does not offer much advantage. What happens next in human development is that we learn to map symbols onto these representations, and then we learn to manipulate those symbols in ways that eventually support our advanced mathematical competencies. We also know that cultural differences, including learning strategies and the way in which reading is performed, can have impacts on how the brain processes some types of numerical information [50], indicating a relation between early experience and numerical processing. Although no one expects that a pigeon or chimpanzee would ever learn trigonometry or calculus, it remains to be seen what greater capacities might one day be exhibited by nonhuman animals. Human children are raised in environments in which numerical information is everywhere, and number words and number symbols are used frequently. One future step should be to provide animals with the type of environment that supports the emergence of more complicated mathematical skills. Longitudinal studies with animals exposed to logically structured, highly enriching environments that focus on numerical development are needed, as are additional cross-cultural studies with humans. Coupled with the emerging capacities for tracking brain activity and the creative methodologies for understanding numerical skills that are outlined above, we should have every expectation of continuing to improve our understanding of the evolutionary and developmental foundations of mathematics.

## Abbreviations

IPS: intraparietal sulcus
PFC: prefrontal cortex
VIP: ventral intraparietal region
